# New Insights into the Genome Organization of Yeast Killer Viruses Based on “Atypical” Killer Strains Characterized by High-Throughput Sequencing

**DOI:** 10.3390/toxins9090292

**Published:** 2017-09-19

**Authors:** Manuel Ramírez, Rocío Velázquez, Antonio López-Piñeiro, Belén Naranjo, Francisco Roig, Carlos Llorens

**Affiliations:** 1 Departamento de Ciencias Biomédicas (Área de Microbiología, Antiguo Rectorado), Facultad de Ciencias, Universidad de Extremadura, Badajoz 06071, Spain; rociovelazquez1981@gmail.com (R.V.); bnaranjo79@gmail.com (B.N.); 2 Departamento de Biología Vegetal, Ecología y Ciencias de la Tierra, Facultad de Ciencias, Universidad de Extremadura, Badajoz 06071, Spain; pineiro@unex.es; 3 Biotechvana, Parc Científic, Universitat de València, Calle Catedrático José Beltrán 2, Paterna 46980 (València), Spain; francisco.roig@uv.es (F.R.); carlos.llorens@biotechvana.com (C.L.)

**Keywords:** *Saccharomyces cerevisiae*, *Torulaspora delbrueckii*, killer, virus genome, dsRNA, sequencing, HTS, RNA recombination, phylogenetic origin

## Abstract

Viral M-dsRNAs encoding yeast killer toxins share similar genomic organization, but no overall sequence identity. The dsRNA full-length sequences of several known M-viruses either have yet to be completed, or they were shorter than estimated by agarose gel electrophoresis. High-throughput sequencing was used to analyze some M-dsRNAs previously sequenced by traditional techniques, and new dsRNAs from atypical killer strains of *Saccharomyces cerevisiae* and *Torulaspora delbrueckii*. All dsRNAs expected to be present in a given yeast strain were reliably detected and sequenced, and the previously-known sequences were confirmed. The few discrepancies between viral variants were mostly located around the central poly(A) region. A continuous sequence of the ScV-M2 genome was obtained for the first time. M1 virus was found for the first time in wine yeasts, coexisting with Mbarr-1 virus in *T. delbrueckii*. Extra 5′- and 3′-sequences were found in all M-genomes. The presence of repeated short sequences in the non-coding 3′-region of most M-genomes indicates that they have a common phylogenetic origin. High identity between amino acid sequences of killer toxins and some unclassified proteins of yeast, bacteria, and wine grapes suggests that killer viruses recruited some sequences from the genome of these organisms, or vice versa, during evolution.

## 1. Introduction

Killer yeast strains secrete protein toxins that are lethal to non-killer strains, and other types of killer strains belonging to the same yeast species. They also can kill other yeast species [1,2]. Each killer yeast is immune to its own toxin and also to the toxins produced by other yeast strains with the same type of killer phenotype [3]. The *Saccharomyces cerevisiae* killer strains have been grouped so far into four types (K1, K2, K28, and Klus) based on their killing profiles and lack of cross-immunity. In general, the killer activity of Klus strains is weaker than that of K1, K2, and K28 strains [2], and they are usually the most difficult to analyze and define with precision. The aforesaid toxins are encoded by the positive strand of medium-size (1.6 to 2.4 kb) dsRNA of yeast viruses (M1, M2, M28, and Mlus, respectively). The RNA 5′-end region contains an ORF that codes for the toxin precursor or preprotoxin (pptox), which also provides immunity to its own killer toxin. The four toxin-coding M dsRNAs show no overall sequence identity with each other or with M dsRNAs of other yeast species [1,2,4]. These M viruses depend on a second large-size (4.6–4.8 kb) dsRNA helper virus, LA, for maintenance and replication. LA provides the capsids that contain the RNA-polymerase, in which both LA and M dsRNAs are separately encapsidated and replicated. The M dsRNAs contain some stem-loop structures (VBS, viral binding site; IRE, internal replication enhancer; and 3′-TRE, 3′-terminal recognition element) that mimic those LA dsRNA signals which are required for genome packaging or replication (reviewed by Schmitt and Breinig, 2006 [3]). There are some other non-*Saccharomyces* killer yeasts containing a similar set of dsRNA helper and satellite viruses responsible for their killer phenotype, such as *Hanseniaspora uvarum*, *Zygosaccharomyces bailii*, *Ustilago maydis*, and *Torulaspora delbrueckii* [1,5,6]. Of these other killer toxins, zygocin from the osmotolerant yeast *Zygosaccharomyces bailii* has been the most studied [7,8,9]. Although M genomes show no overall sequence homology, they share a very similar genomic organization. Additionally, a relevant identity of some regions of *T. delbrueckii* Mbarr-1 genome with the putative replication and packaging signals of most of the M-virus RNAs suggests that they are all evolutionarily related [1,2]. To date, the presence of more than one killer virus in a single yeast cell has not been reported. The M-dsRNAs from different viruses are believed to exclude each other at the replicative level, although the mechanism of this exclusion is still unknown [3,10]. This explains why no more than one type of killer virus has been found so far in a single yeast cell.

The LBC virus of *S. cerevisiae* is an LA-related virus, with a similar genome size, which coexists with LA in many killer and non-killer *S. cerevisiae* strains [11,12]. LBC shows no relevant overall sequence homology with LA (except in the functional domains of RNA-polymerases), and has no known helper activity. However, LA and LBC share the same genomic organization, coding for two proteins—the major coat protein Gag and a minor Gag-Pol fusion protein translated by a -1 ribosomal frameshifting mechanism, which contains all the activities required for virus propagation [13,14,15,16].

The cis signals required for RNA packaging and replication are located in the 3′-terminal regions of the positive strands of both LA and M RNAs [2,17]. The signal for transcription initiation of the mRNA (positive strand) has been proposed to be present in the 3′-end first 25 nucleotides of the LA RNA negative strand, probably in the very 3′-terminal sequence itself (3′-CTTTTT, 5′-GAAAAA in the positive strand). This 3′-terminal recognition element is also present in the 3′ end of M1, M2, M28, and Mlus RNAs [2,18,19,20].

The ORF of M1, M2, or M28 is translated into a preprotoxin that subsequently enters the secretory pathway for further processing and secretion as a mature toxin. The unprocessed preprotoxin (pptox) consists of an N-terminal signal sequence necessary for its import into the endoplasmic reticulum lumen, followed by the α- and β-subunits of the mature toxin separated from each other in the cases of K1 and K28 by a potentially *N*-glycosylated γ-sequence. The signal peptide is removed in the endoplasmic reticulum, and *N*-glycosylation and disulfide bond formation occurs. Then, in a late Golgi compartment, protease processing takes place involving Kex2 and Kex1 proteases. Finally, the toxin is secreted as an active α/β heterodimer, with the two subunits being covalently linked by one or more disulfide bonds [3,17]. All this processing is also believed to occur in Klus (*S. cerevisiae*) and Kbarr-1 (*Torulaspora delbrueckii*) preprotoxins in accordance with their predicted amino acid sequences [1,2].

Several dsRNA genomes of LA and M viruses have already been sequenced, although the sequences of some viruses such as that of ScV-M2 [21] have yet to be completed. Additionally, the known sequences of some viruses, including ScV-Mlus (2033 nucleotides), are shorter than estimated by agarose gel electrophoresis (2.3 kb). This difference has been explained as being due to a variable number of adenine residues in the central A-rich region, which supposedly also accounts for the different sizes of ScV-Mlus isotypes. This central A-rich region may facilitate sliding or jumping of either the reverse transcriptase or the Taq polymerase used in RT-PCR prior to sequencing, yielding a slightly shorter sequence than the actual one [2]. Besides this, although cDNA clones of LA have been used extensively to analyze this virus’s biology, the launching of LA virus from transcripts of the cloned cDNA to yeasts lacking this virus has not yet been possible [22]. All these circumstances taken together lead us to suspect the presence of extra nucleotide sequences beyond those motifs to date accepted as the ends of these virus genomes, which still remain unknown. These circumstances very much limit the detailed analysis of these viruses’ genomes and their biological behavior.

The aim of the present work was to: (i) use high-throughput sequencing (HTS) to re-characterize some M viruses (from *S. cerevisiae* and *T. delbrueckii* wine yeasts) that were previously sequenced by traditional techniques of cloning and sequencing, to confirm the already-known sequence and search for extra sequences beyond the ends of the genome; (ii) sequence new M-virus dsRNA isotypes with electrophoretic migration faster than usual, or infecting killer yeasts showing atypical killer phenotype; and (iii) analyze the newly obtained sequences to look for putative functional domains and phylogenetic relationships between these killer viruses. This strategy has allowed us to extend the genome sequence of these killer viruses to provide new insight into their relationship with the hosting yeast cell and the helper L viruses. The possible evolutionary relationship between these viral M dsRNAs will also be discussed in light of the new findings.

## 2. Results

### 2.1. Phenotypic and Genotypic Characterization of S. cerevisiae and T. delbrueckii Killer Yeasts

Killer phenotype analysis of *S. cerevisiae* killer strain EX1125 revealed that it behaved as a typical K2 yeast. It killed non-killer (sensitive), K1, and K28 strains of *S. cerevisiae* in at least one of the four killer-plate assay conditions. *S. cerevisiae* EX231 was originally considered K2 because it did not kill K2 strains, and no K1 yeast had previously been found in wine-related environments; however, it showed atypical K2 phenotype because it did not kill K1 strains. This indicates that EX231 could actually be K1 yeast. *S. cerevisiae* EX229 and EX436 were typical Klus yeasts, able to kill sensitive, K1, K2, and K28 strains of *S. cerevisiae* [2]. EX1160 was originally considered to be a K2 strain with weak killer activity. However, it was never killed by K2 strains, and it eventually killed some K2 strains, which indicates that it could actually be a Klus yeast. *T. delbrueckii* EX1180 was a typical Kbarr-1 strain that killed the four known killer types of *S. cerevisiae* (K1, K2, K28, and Klus) and the *T. delbrueckii* Kbarr-2 EX1257 strain [1]. However, this Kbarr-2 EX1257 strain did not kill Kbarr-1 strains. It only showed weak killer activity against non-killer and K2 killer strains of *S. cerevisiae* [1], and moderate killer activity against a non-killer (Kbarr-1^0^) strain obtained from *T. delbrueckii* EX1180, EX1180-2K^−^ (Figure S1).

**Figure 1:**
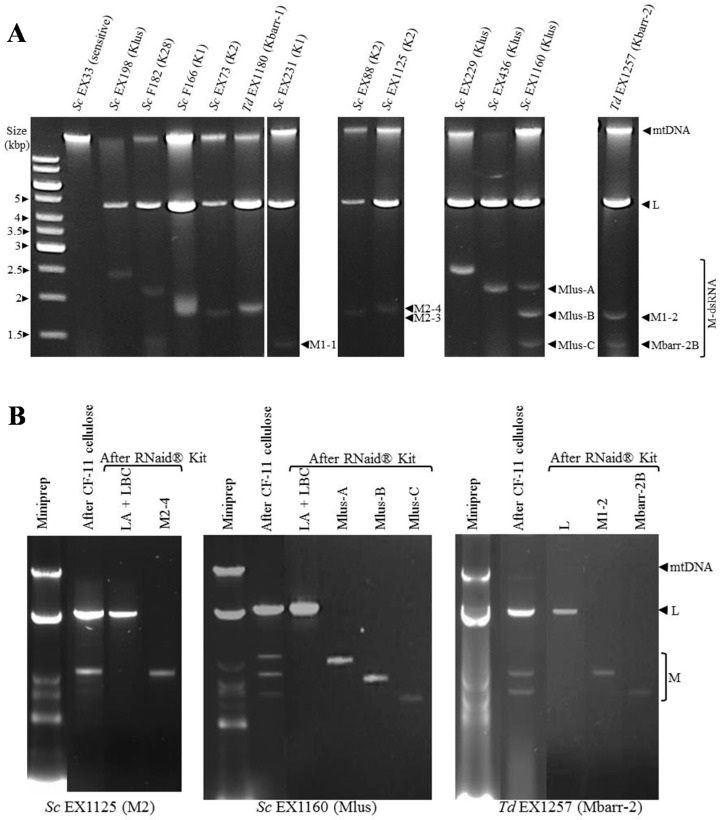
Genetic determinants of killer phenotype. (**A**) Presence of viral dsRNA molecules in killer strains. Nucleic acids were obtained from sensitive (EX33), K1 (F166, EX231), K2 (EX73, EX88, EX1125), K28 (F182), Klus (EX198, EX436, EX229, EX1160), Kbarr-1 (EX1180), and Kbarr-2 (EX1257) strains, and separated by agarose gel electrophoresis. The name of each viral genome isotype is shown on the right of each dsRNA. *Sc*, *Saccharomyces cerevisiae*. *Td*, *Torulaspora delbrueckii*; (**B**) Purification of dsRNA viral genomes from EX1125 (K2), EX1160 (Klus), and EX1257 (Kbarr-2). Samples from each purification stage were separated by agarose gel electrophoresis. The ethidium bromide staining of the gel is shown.

All killer strains contained at least two nucleic acid molecules with agarose-gel-electrophoresis migration similar to viral dsRNAs from other killer yeasts: (1) a slower-moving band, similar in size to the dsRNA genome of ScV-LA and ScV-LBC viruses (4.6–4.8 kb) named TdV-LA and TdV-LBC for *T. delbrueckii*; and (2) one to three faster-moving bands, similar to the dsRNA genome of ScV-M or TdV-M viruses (1.3–2.3 kb). *S. cerevisiae* EX1160 contained three M bands (Mlus-A, Mlus-B, and Mlus-C), all with high sequence identity with the previously-known Mlus-4 virus genome (see below). Surprisingly, *T. delbrueckii* EX1257 contained two M bands, M1-2 and Mbarr-2B, which showed high sequence identity with two different M-viruses, M1 and Mbarr-1, respectively (see below). The rest of the strains seemed typical killer yeasts containing only one M band (Figure 1A, Table 1). The dsRNA nature of these nucleic acid bands was confirmed by DNAse I and RNAse A treatments, as previously described [1,2]. As expected, the mtDNA band disappeared after DNAse I treatment, while L and M bands remained unaffected. However, L and M bands disappeared after RNAse A treatment, while the mtDNA band remained unaffected. Additionally, L and M bands were fairly resistant to RNAse A digestion in the presence of 0.5 M NaCl. The same results were obtained for all the killer yeasts analysed (data not shown), which indicates that L and M bands were dsRNA, but not ssRNA [23,24,25]. All killer strains lost the M dsRNA bands after growing in the presence of cycloheximide, and a concomitant loss of their killer activity was observed. This result indicates that their killer phenotype is encoded by the M dsRNA, as has previously been described for other killer toxins encoded by M1, M2, M28, and Mlus dsRNAs in *S. cerevisiae* [2,23,24,25,26,27] or Mbarr-1 in *T. delbrueckii* [1]. Despite this, these non-killer yeasts often recovered the killer phenotype and the corresponding M dsRNA band after 20–30 doublings (4–6 transfers) in YEPD plates at 30 °C (usual laboratory propagation conditions). In fact, after more than 20 attempts, we failed to get stable non-killer yeasts from the EX1257 killer strain (Figure S1).

### 2.2. Analysis of the dsRNA Sequence from ScV-M and TdV-M Viruses

The dsRNA bands from each killer yeast were purified (Figure 1B) and sequenced by HTS techniques. Sequences belonging to already-known yeast viruses were found in all dsRNA bands. In some cases, the complete sequence obtained was longer than the size estimated by agarose gel electrophoresis, and longer than the previously-known sequence for each virus (Table 1). For sequence description, nucleotides were numbered from the 5′GAAAAA conserved motif, which is generally accepted as the 5′-end in most viral L and M genomes, and probably required for transcription initiation [28]. The 5′-terminal G was denoted as number 1. Extra nucleotides found upstream from the 5′GAAAAA motif were numbered with a negative symbol starting at (−)1 from the first nucleotide upstream from 5′-G. Extra nucleotides found downstream from the previously considered as 3′-end were numbered with a positive symbol starting at (+)1 from the first nucleotide located downstream.

M1 genome was found in wine yeasts for the first time in *S. cerevisiae* EX231, as well as in *T. delbrueckii* EX1257. These findings were confirmed by qPCR using specific primers targeting the K1 toxin coding sequence, as well as the 5′-extra sequences obtained by HTS.

**Figure 2:**
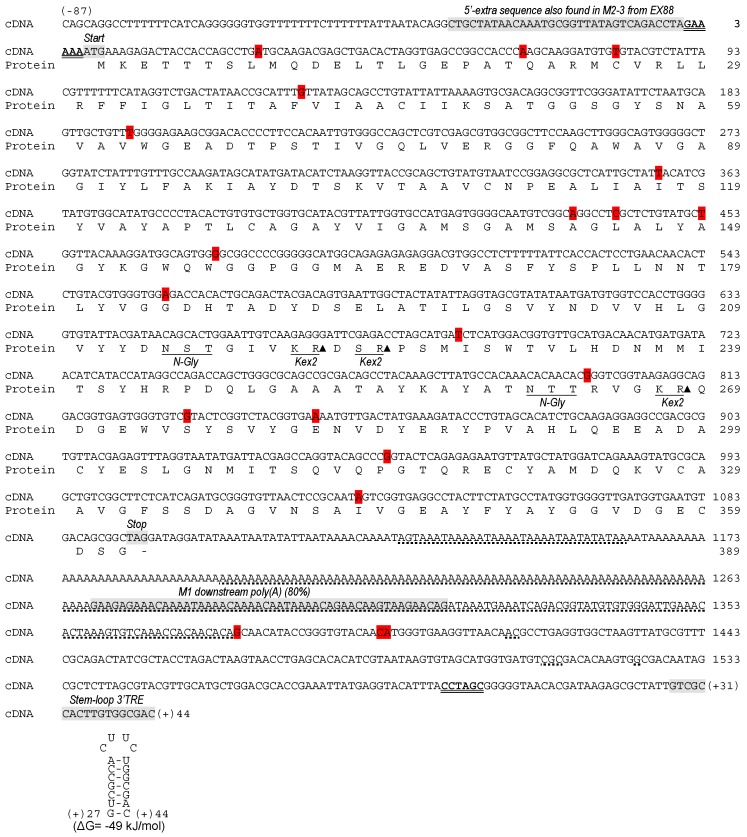
Nucleotide sequence of the ScV-M2-4 genome (cDNA) and amino acid sequence of the putative ORF of K2 preprotoxin. The amino acid sequence is displayed under the nucleotide sequence. The previously known 5′ and 3′ ends (5′-GAAAAA and CCTAGC-3′, respectively) are in bold face and double underlined. The 5′-extra sequence found in M2-3 and M2-4, protein synthesis initiation codon (start), protein synthesis stop codon (stop), putative 3′-terminal recognition element for virus replication (stem-loop 3′TRE, with a free energy of ΔG = −49 kJ/mol), and a region 80% identical to ScV-M1 dsRNA located downstream from the central poly(A) [M1 downstream poly(A)] are shown grey shaded in the nucleotide sequence. Sequence missing in the previously-known sequencing of M2 from *S. cerevisiae* 1384 strain is dot underlined. Nucleotides changed in M2-4 with respect to M2-genomes from strains 1384 and EX88 are shown red shaded. The putative Kex2 endopeptidase sites and potential *N*-glycosylation sites (N-Gly) are underlined in the amino acid sequence. Closed triangles indicate cleavage site. The secondary structure of the putative cis signal for replication of M2 virus is at the bottom of the sequence.

A continuous sequence of M2 genome was obtained for the first time (GenBank accession number MF957266). It was obtained twice, from two different K2 strains, EX1125 and EX88. Both M2 genomes contained the sequence of two gaps that were missing in the previously-known M2 sequence from yeast strain 1384 [21,29,30]. One gap (182 nt) was located downstream from the central poly(A) and contained the greatest part of this poly(A) and the nearest downstream sequence. The other gap (32 nt) was located upstream from the central poly(A). Additionally, 87 and 44 extra nucleotides were found in M2-4 genome from EX1125, up and downstream from the 5′ and 3′ ends previously determined by conventional sequencing, respectively. A putative cis signal for replication (3′-TRE) for M2 virus was now found in M2-4 (Figure 2). An extra stretch of 33 nucleotides was found in M2-3 genome from EX88 upstream from the 5′ end, which is 100% identical to the same stretch of M2-4. This makes a total length of 1723 nt for M2-4 and 1625 nt for M2-3; figures which are in agreement with the lengths estimated by agarose-gel electrophoresis—1750 bp and 1650 bp, respectively. Also, these two M2 sequence sizes are more in agreement with the previously estimated length for M2 from the 1384 strain (1700 bp) than with the published original sequence from the same strain, 1163 + 209 nt [21,29,30] (Table 1, Figure 2).

Similarly, the length of Mlus-4 genome from the EX229 strain (2314 nt) was in agreement with the size estimated by agarose-gel electrophoresis (2300 bp). Some extra nucleotides were also found up and downstream from the 5′ and 3′ ends (50 and 230 nt, respectively) previously determined by conventional sequencing (NCBI/GenBank accession number GU723494, [2]), where around 300 nt were missing.

For the rest of the viral genomes analyzed, the sequences obtained were longer than the length estimated by gel electrophoresis. The size differences ranged from 168 in Mlus-A to 705 bp in Mlus-C. Moreover, surprisingly, the sequences of these viruses were even longer than the sequences of other dsRNA isotypes belonging to the same virus type, but showing slower mobility in gel electrophoresis. This was the case for: M1-1 from EX231 (1933 bp) and M1-2 from EX1257 (1933 bp) with respect to the previously-known M1 from TF325 strain (1801 bp); Mlus-1 from EX436 (2346 bp), Mlus-A (2268 bp) from EX1160, and Mlus-C (2055 bp) from EX1160 with respect to Mlus-4 from EX229 (2033 bp); and Mbarr-2B from EX1257 (1835 bp) with respect to Mbarr-1 from EX1180 (1705 bp) (Table 1). These results indicate that the presence of extra sequences at the ends of the viral dsRNA might change its tridimensional conformation, making it migrate faster than expected in agarose-gel electrophoresis in native conditions. In fact, several 5′- and 3′-terminal sequences of these viruses can form single-strand stem-loop structures with very negative ΔG, from −30.1 to −205 kJ/mol (Table 1). Taking this into primary consideration, we would speculate that the formation of these structures in each RNA strand may change the tridimensional organization of these dsRNA genomes to form a more globular molecule, able to migrate faster than the linear form of dsRNA in agarose-gel electrophoresis.

### 2.3. Analysis of 5′- and 3′-Extra Sequences of M-Genomes

No relevant overall identity was found between 5′- and/or 3′-extra sequences of the different viruses. Only some local identity was found among the 5′-extra sequence of some genomes belonging to the same virus killer type: a 33 nt stretch [A(−)1 to C(−)33] 100% identical in M2-3 from EX88 and M2-4 from EX1125 (Figure 2); and a 21 nt stretch [5′-CGTAACTAAGTAAGTGATAGT-3′] 100% identical in Mlus-4 from EX229, Mlus-1 from EX436, and Mlus-A from EX1160. Additionally, a poly(G) stretch was found in the 5′-extra sequence of both Mbarr-2B (10-nt) and M1 (14-nt), both from the same *T. delbrueckii* EX1257 strain, although they are different virus types (data not shown).

However, the 3′-extra sequence of four M-viruses contained stretches highly identical to ribosomal RNA sequences: M1-2 from EX1257 (a 77 nt stretch 94% identical to *S. cerevisiae* 26S rRNA), Mlus-4 from EX229 (a 242 nt stretch 98% identical to *S. cerevisiae* 16S mitochondrial rRNA), Mlus-1 from EX436 (a 198 nt stretch 100% identical to *S. cerevisiae* 18S rRNA), and Mbarr-2B from EX1257 (a 76 nt stretch 97% identical to *T. delbrueckii* 26S rRNA), (Table 1). None of these sequences share relevant identities—not even the 26S-rRNA stretches found in M1-2 and Mbarr-2B that coexisted in the same yeast strain (EX1257), i.e., these sequence stretches belong to different parts of the 26S-rRNA. With respect to the 5′-extra sequences, M1-1 from EX231 contained a 66 nt stretch 94% identical to *S. cerevisiae* LBC-2 virus, Mlus-1 from EX436 contained a 47 nt stretch 93% identical to a genomic sequence of *Vitis vinifera* (wine grape) that includes another 45 nt stretch 85% identical to a genomic sequence of *Saccharomycopsis fibuligera*, and Mbarr-2B from EX1257 contained a 41 nt stretch 88% identical to a genomic sequence of *Cucumis melo* (melon). No similar sequence identity was found for the 5′- or 3′-extra sequences of M2 viruses (Table 1).

### 2.4. Analysis of Common Core Sequence of M-Genomes

Almost no difference (>99% identity) was found in the common core sequence (without considering the newly found extra sequences) of the three M1 genomes, and their toxin coding regions were almost identical. The M1-2 genome from EX1257 strain contained an extra T inserted in the central poly(A), position 1110, with respect to the original M1 from TF325 strain. M1-1 from EX231 strain contained 15 single-nucleotide changes, 13 Gs and 2 Cs, scattered in the A-rich region located immediately downstream of the central poly(A), which were As in the original M1 from the TF325 strain.

The common core sequence of the two Mbarr genomes, Mbarr-1 and Mbarr-2B, were identical. The only difference between them was that Mbarr-2 had extra sequences at both ends with respect to Mbarr-1.

The three M2 genomes also shared a high degree of identity in the common core sequence. As mentioned above for M2-4 from EX1125, M2-3 from EX88 contained two internal fragments that were missing in the two partial sequences of M2 from the 1384 strain [21,29], a 32-nt stretch upstream of central poly(A), and a 183-nt stretch containing most of central poly(A) and the immediate downstream region (where a region 80% identical to ScV-M1 dsRNA was found, Figure 2). Besides this, in the killer toxin coding region and with respect to the original sequence of M2 from the 1384 strain, there were 16 single-nucleotide changes in M2-4 from EX1125, eight of which produced amino acid changes (31G > A [amino acid change 9V > M], 68G > A [21R > Q], 81C > T, 127A > G [41I > V], 193C > T [63R > W], 357C > T, 435G > A, 441T > C, 453C > T, 475A > G [157S > G], 558G > A, 689C > T [228T > I], 831A > G, 849G > A, 949T > G [315C > G], and G1035 > A [343M > I]), and just three changes in M2-3 from EX88, two of which produced amino acid changes (689C > T [228T > I], 797A > G [R > 264Q], and 831A > G). Additionally, in the non-coding 3′-half of the sequence, two single nucleotide changes were found in M2-4 from EX1125 (C1378G, G1398A), and another two in M2-3 from EX88 (C1378G, C1397T). Moreover, nucleotides A1416, C1417, C1511, G1512, C1513, G1524 found in M2-3 and M2-4 were missing in the original M2 from the 1384 strain. Nevertheless, none of these changes seem to be relevant, since they do not affect important amino acids of K2 toxin or relevant domains of the M2 genome (Figure 2).

The Mlus-4 sequences from EX229 (both the original sequence obtained by conventional techniques and the new one obtained by HTS), Mlus-1 from EX436, and Mlus-C from EX1160 were also almost 100% identical. All four contained the two central poly(A)s [5′- and 3′-poly(A)] previously described in Mlus-4 [2], and shared the same sequences on both sides (up and downstream) and in-between these two poly(A)s. They only differed in some scattered nucleotides in the two central poly(A)s, and in that Mlus-1 contained a 64-nucleotide A+G-rich stretch inserted in position 976, just downstream of the 5′-poly(A). Mlus-A and Mlus-B from EX1160 contained as many as 68 nucleotide changes scattered up and downstream from the central region containing the two poly(A)s, most (54 changes) identical in both genomes, and a common 1145A > C change in the stretch located between the two central-poly(A)s. Additionally, two stretches were missing in Mlus-B, one of 109 nt that includes the 3′-poly(A) (from G1169 to G1277), and another of 22 nt located roughly in the middle of the non-coding 3′-half of the genome (from A1626 to T1647). Mutations in the toxin-coding region of Mlus-A and Mlus-B imply 11 single amino acid changes with respect to the original sequence of Mlus-4 from EX229, nine identical in both genomes (5S > G, 9C > Y, 38Y > V, 89V > I, 137S > A, 139D > N, 167K > E, 205V > I, and 232S > G), one specific in Mlus-A (33H > R), and another one specific in Mlus-B (166V > A). Again, mostly these changes do not seem to be relevant, as they affect no important amino acid for Klus toxin processing, except maybe the change 33H > R in Mlus-A located in the first putative *N*-glycosylation site [2], which could produce a different glycosylation pattern than Mlus-4 from EX229.

**Figure 3:**
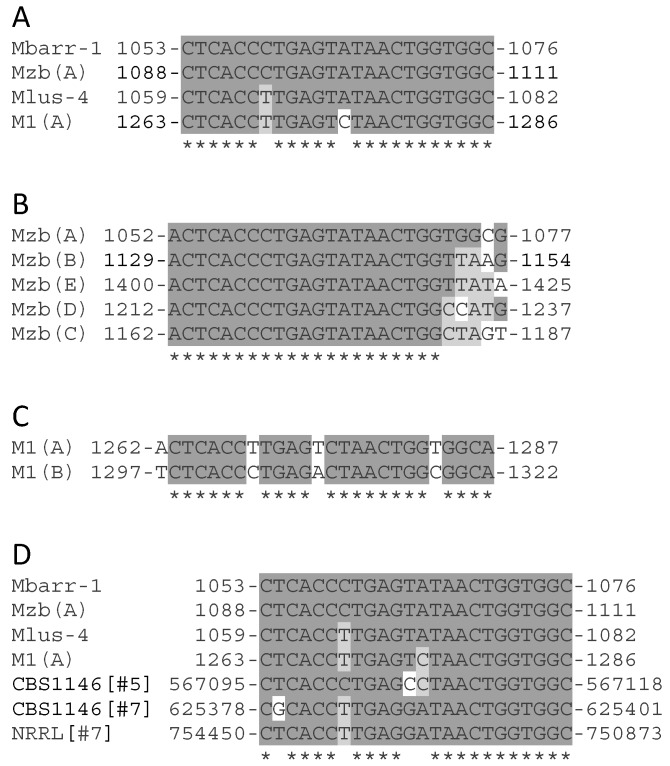
Relationship between the 24-nt motifs found in the non-coding 3′-region of M-viruses (M1, Mlus, Mbarr-1, and Mzb; GenBank accession number U78817.1, GU723494, KT429819, and AF515592.1, respectively) and chromosome sequences of several yeast strains. (**A**) Comparison of the nucleotide sequences of 24-nt motif of all viruses (one from each virus); (**B**) Comparison of the five copies of 24-nt motif found in Mzb; (**C**) Comparison of the two copies of 24-nt motif found in M1 (the same copies were found in M1-1 and M1-2 isotypes); (**D**) Comparison of one copy of 24-nt motif from each virus with the same motif found in chromosome #5 of *T. delbrueckii* CBS 1146 (CBS1146[#5], GenBank accession number HE616746.1), chromosome #7 of *T. delbrueckii* CBS 1146 (CBS1146[#7], GenBank accession number HE616748.1), and chromosome #7 of *T. delbrueckii* NRRL Y-50541 (NRRL[#7], GenBank accession number CP011784.1). The comparison was done using the ClustalW multiple sequence alignment program. Asterisks (*) indicate identical nucleotides. Nucleotides identical to those of the first sequence (top of each figure) are dark-grey shaded, and the ones identical among some of the other sequences are light-grey shaded.

Although the dsRNAs of all known types of M-viruses show no overall sequence identity with each other [1,2,4], relevant identity between some sequence stretches of TdV-Mbarr-1 RNA and some M-RNAs from *Saccharomyces* (ScV-M1, ScV-M28) and *Zygosaccharomyces bailii* (ZbV-Mzb) have been found previously. This raised the possibility that M viruses of *T. delbrueckii*, *S. cerevisiae*, and *Z. bailii* could have a common phylogenetic origin, at least for the non-coding 3′-region where these homologous sequences were located [1]. In this sense, we found a new 24-nt motif in the respective non-coding 3′-regions of M1, Mlus, Mbarr, and Mzb viruses, which share 92–100% identity (Figure 3A). This motif was repeated five times in Mzb (A to E) and twice in M1 (A to B) genomes (Figure 3B,C), and it is also present in the genomic DNA of two *T. delbrueckii* strains recently sequenced (in chromosomes #5 and #7 of the CBS 1146 strain, and chromosome #7 of the NRRL Y-50541 strain) (Figure 3D).

**Figure 4:**
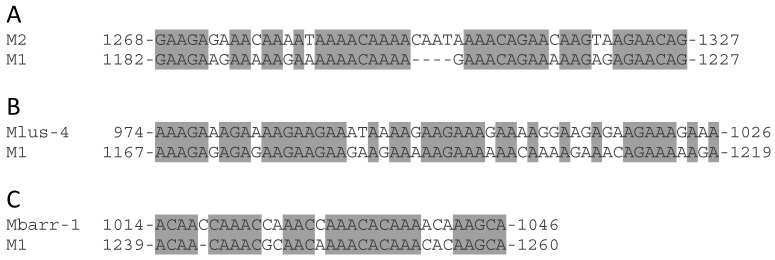
Local identity found in the non-coding 3′-region of M-viruses, between central poly(A) and 24-nt motifs. (**A**) Comparison of the nucleotide sequences of M2 (GenBank accession number MF957266) and M1; (**B**) Mlus-4 and M1; (**C**) Mbarr-1 and M1. Identical nucleotides are dark-grey shaded.

This 24-nt motif was found neither in the M2 and M28 genomes, nor in *S. cerevisiae* or *S. paradoxus* genomic DNA which can be infected by M1 and Mlus viruses. However, a similar stretch sharing from 83% to 90% identity was found in other *Saccharomyces* yeasts (i.e., *S. bayanus* 623-6C YM4911, *S. bayanus* MCYC 623, *S. pastorianus* CCY48, *S. kudriavzevii* IFO 1803, *S. kudriavzevii* IFO 1802, and *S. pastorianus* Weihenstephan 34/70), and a less similar stretch sharing 75% identity in *Zygosaccharomyces bailii* ISA1307 WGS strain (data not shown). Besides this, some local identity was found in the region located between the central poly(A) and the 24-nt motifs for M2 and M1 (80% identity), Mlus and M1 (70%), and Mbarr and M1 (81%) (Figure 4). These results suggest that some recombination events could have occurred between different M viruses in this region downstream from the central poly(A).

Some stretches located in the non-coding 3′-half of Mlus and Mbarr viruses, downstream from the 24-nt motifs, also shared relevant identity with genomic sequences of other organisms. All Mlus viruses contained a 42 nt stretch [T1234 to A1275] 85% identical to a genomic sequence of *Vitis vinifera*, and a 29 nt stretch [A1634 to T1662] 93% identical to a genomic sequence of *Candida glabrata*. Mbarr-1 and Mbarr-2B contained a 38 nt stretch [A1049 to G1086] 90% identical to a sequence of chromosome #7 of *Torulaspora delbrueckii* NRRL Y-5054. These results suggest that there probably was some input of RNA sequences from yeast or wine grape genome ORFs into this region.

### 2.5. Analysis of ScV-M and TdV-M Preprotoxin ORF Sequences

**Figure 5:**
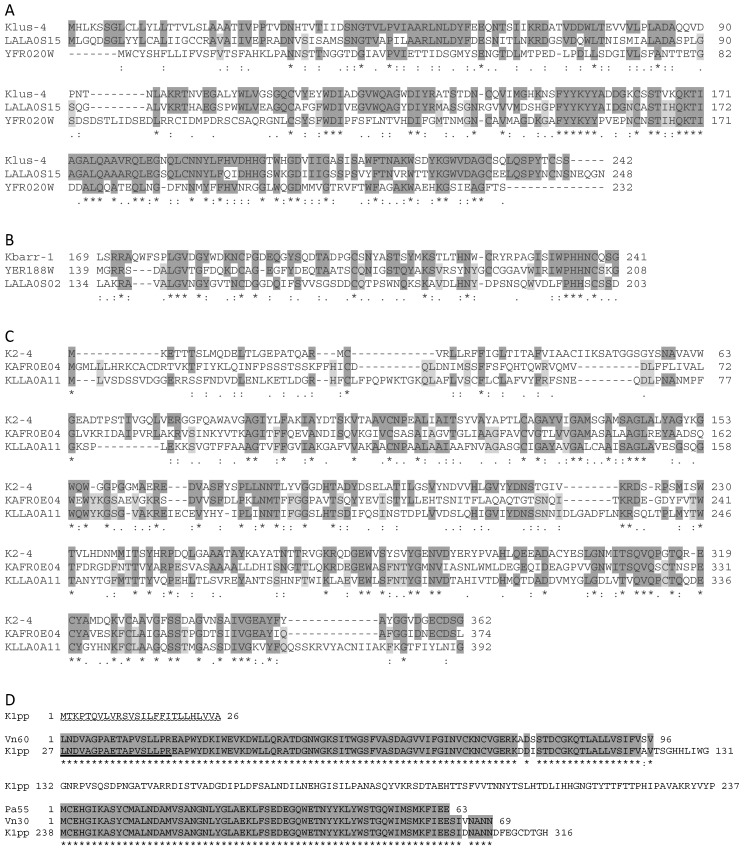
Relevant identity found between the amino acid sequence of M-virus preprotoxins and some hypothetical proteins (ORFs) of bacteria and yeasts. (**A**) Klus-4 preprotoxin and two hypothetical proteins: LALA0S15e00254g1_1 from *Lachancea lanzarotensis* (shown as LALA0S15), and YFR020W from *S. cerevisiae* (YFR020W); (**B**) A stretch of Kbarr-1 preprotoxin and two hypothetical protein stretches: YER188W from *S. cerevisiae* S288c (YER188W), and LALA0S02e11166g1_1 from *L. lanzarotensis* (LALA0S02). (C) K2-4 preprotoxin and two hypothetical proteins: KAFR_0E04500 from *Kazachstania africana* CBS 2517 (KAFR0E04), and KLLA0A11979p from *K. lactis* NRRL Y-1140 (KLLA0A11). (D) K1 preprotoxin (K1pp) and three hypothetical proteins: AKJ17_18960 from *Vibrio nereis* (Vn69), ABT56_23155 from *Photobacterium aquae* (Pa55), and AKJ17_18930 from *V. nereis* (Vn30). The amino-terminal signal peptide (26 amino acid residues) and the pro-region (18 residues) are wavy underlined and underlined, respectively. The comparison was done using the ClustalW multiple sequence alignment program. Asterisks (*) indicate identical amino acids; double dots (:) and single dots (.) indicate conserved and semi-conserved substitutions of amino acids, respectively. Amino acids identical to those of the toxin protein (top of each figure) are dark-grey shaded, and the ones identical among the other proteins are light-grey shaded.

According to the predicted amino acid sequences of all the killer toxins’ ORFs, they seem to be translated as preprotoxins, processed, and secreted as previously described for K1 and K28 killer toxins of *S. cerevisiae* [3,17]. However, their amino acid sequences did not share any relevant overall or local homology. Nonetheless, some relevant identity was found between these toxins’ sequences and the amino acid sequence of some ORFs from yeasts and bacteria. As previously described, the Mlus ORF-encoded protein showed 32% identity with an *S. cerevisiae* chromosomally encoded ORF protein of 232 amino acids (YFR020W) of unknown function. This identity increased to 44% when only the C-terminal half of the two proteins was considered [2]. Additionally, we found that Mlus ORF protein showed as much as 57% identity with a genomic *Lachancea lanzarotensis* ORF of 248 amino acids (LALA0S15e00254g1_1). This identity also increased to 67% when only the C-terminal half of the two proteins was considered (Figure 5A).

Mbarr ORF-encoded protein showed 45% identity with an *S. cerevisiae* (S288C strain) chromosomally encoded ORF protein of 239 amino acids (YER188W) of unknown function. It also showed 31% identity with a genomic *Lachancea lanzarotensis* ORF of 227 amino acids (LALA0S02e11166g1_1). These relevant identities were also found when only the C-terminal half of the three proteins was considered (Figure 5B).

The M2 ORF-encoded protein showed 32% identity with a *Kazachstania africana* (CBS 2517 strain) chromosomally encoded ORF protein of 374 amino acids (KAFR_0E04500), and 35% identity with a *Kluyveromyces lactis* (NRRL Y-1140 strain) chromosomally encoded ORF protein of 392 amino acids (KLLA0A11979p), both of unknown function. Again, these identities became more relevant when only the central and C-terminal portions of the three proteins were considered (Figure 5C).

In contrast to aforementioned M viruses, M1 preprotoxin showed the greatest homology with hypothetical proteins from bacteria instead of yeasts. The N-terminal stretch of K1 pro-toxin showed 99% identity with a *Vibrio nereis* hypothetical protein (AKJ17_18960, 96 amino acids). Additionally, the C-terminal stretch of K1 toxin was 96% identical to a *Vibrio nereis* hypothetical protein (AKJ17_18930, 70 amino acids), and 100% identical to a *Photobacterium aquae* hypothetical protein (ABT56_23155, 63 amino acids) (Figure 5D). These results again suggest that there probably was some ancient input of RNA sequences from yeast or bacteria into the toxin’s ORF in the process of emergence of each M-virus type.

## 3. Discussion

### 3.1. Phenotypic and Genotypic Characterization of Atypical Killer Yeasts

The atypical killer yeasts analyzed in this study contained M-viruses with different dsRNA sequence or tridimensional structure than typical killer yeasts of the same type. This indicated that atypical viruses may contain some dsRNA domains responsible for the atypical killer phenotype of some killer yeasts. Besides this, some of these viruses can apparently be cured from the hosting yeast, but they reappear after several yeast population doublings. These viruses could contain some specific genomic features that allow them to get safely stored somewhere in the yeast cell. This ability would ensure their maintenance in the hosting yeast, and could explain why they reappear in the cured non-killer yeasts after several doublings.

### 3.2. Analysis of dsRNA Sequences from M Viruses

The expected viruses were found in each dsRNA band purified from previously-known killer yeasts, and unexpected viruses were not detected. As an additional example, LA and LBC were found in the dsRNA L band from EX229, while only LA was found in the equivalent band from EX436 (data not shown), as was to be expected according to a previous study of these two yeasts [2]. This indicates that our procedure for dsRNA purification and HTS is reliable enough to specifically detect the presence or absence of killer viruses in yeasts. Besides this, M1 virus was found for the first time in some of the new wine yeasts analyzed, and, surprisingly, it coexisted with Mbarr-1 virus in *T. delbrueckii* EX1257. This finding really merits further study given that M-dsRNAs from different viruses are believed to exclude each other in the same yeast.

The size of the newly obtained continuous sequence of M2-4 dsRNA from EX1125 is in agreement with the length estimated by gel electrophoresis, and contains the sequence of two gaps that were missing in the previously-known M2-sequence from the 1384 strain [21,29,30]. Although we still cannot fully discard the potential existence of some extra nucleotides beyond the newly known ends of the M2-4 genome, as can be the case for any known yeast virus, a continuous sequence from all *S. cerevisiae* killer virus types is now available for their comparison. The genome sequence of M2-3 from EX88 is almost identical to that of M2-4, and its size is also in agreement with the length estimated by electrophoresis. Similarly, the new HTS-sequence of Mlus-4 from the EX229 strain (2314 nt) is in agreement with the size estimated by electrophoresis (2300 nt) (Table 1), while about 300 bp were missing in the previous published sequence [2]. Notwithstanding these genome size agreements, HTS revealed the presence of extra sequences beyond the previously defined 5′ and 3′ ends—the 5′GAAAA motif and the 3′-TRE (3′-terminal recognition element) stem-loop, respectively—that have been found in all known M viruses. Additionally, the viral genomes present in atypical killer yeasts were longer than estimated, and, surprisingly, some were even longer than other dsRNA isotypes supposedly of greater length. Structure fold analyses revealed that M ssRNAs can be highly structured molecules, which may become even more structured when they contain the extra sequences at the 5′ and 3′ ends. Some of these extra sequences can form single-strand stem-loops with very negative ΔG (−30.1 to −205 kJ/mol) in the M ssRNAs (Table 1). The presence of different extra sequences can be a source of conformational heterogeneity among M dsRNA molecules, which contain a "bubble" of unpaired sequences in the middle of the molecule, as has been observed with electron microscopy [31]. Therefore, the different M dsRNA isotypes may be different stable conformers, sharing most of the same primary structure (the common core sequence), but having different tridimensional conformations [20]. These different conformations could be mainly due to the presence of 5′- and 3′-extra sequences. Some M-dsRNA isotypes may have a conformation that is more compact than other isotypes, making them migrate faster than expected in gel electrophoresis under native conditions, as may be the case for M1-1 and M1-2 (Table 1, Figure 1).

Only some local identity was found in the 5′-extra sequence of some viruses. However, the 3′-extra sequence of four viruses contained stretches highly identical (94–100%) to different ribosomal RNA sequences, and the 5′-extra sequence of three viruses contained stretches highly identical (88–94%) to *S. cerevisiae* LBC-2 virus, to a genomic sequence of wine grape and *Saccharomycopsis fibuligera*, and to a genomic sequence of melon. These results suggest that M-virus RNA could recombine with yeast cell RNA, or RNA present in the growing media (grape or melon juice), and eventually keep part of them bound to the ends of the viral genome to yield new virus isotypes. It seems that M-RNA is able to covalently join some other viral or cellular RNAs in somehow promiscuous ways, as previously suggested for fragments of poliovirus RNA [32] and plant viruses [33]. Using this ability, M viruses could become integrated in cell RNA, as do retroviruses and retrotransposons in chromosomal DNA, allowing them to eventually stay protected under stressing environmental conditions as long as the protecting RNA is not degraded. This strategy could explain why some M viruses reappeared in the cycloheximide-cured non-killer yeasts after several doublings in cycloheximide-free medium, returning the yeast cells to their original killer phenotype.

None or just a few nucleotide changes were found in the common core sequence of the different isotypes for each M-virus type. Moreover, the changes that were found do not seem to be relevant for virus replication or killer toxin processing and secretion. This sequence conservation was somehow unexpected given that RNA viruses are known to undergo rapid genetic change due to the high error frequency of RNA synthesis [34,35]. However, RNA viruses that contain segmented genomes, somewhat similar to the association of L and M yeast viruses, can undergo genetic evolution by re-assortment of the RNA segments. Also, RNA recombination involves the exchange of genetic information between non-segmented RNAs (reviewed by Lai 1992 [36]). Therefore, a possible explanation for our results could be that, although RNA recombination can mediate the rearrangement of viral genes and the acquisition of nonself sequences, it can also mediate the repair of virus mutations, as has previously been suggested [33]. In this way, the maintenance of some functional copies of the original M virus could be ensured, which in the long term would maintain almost invariable the common functional sequence of the different virus isotypes.

**Figure 6:**
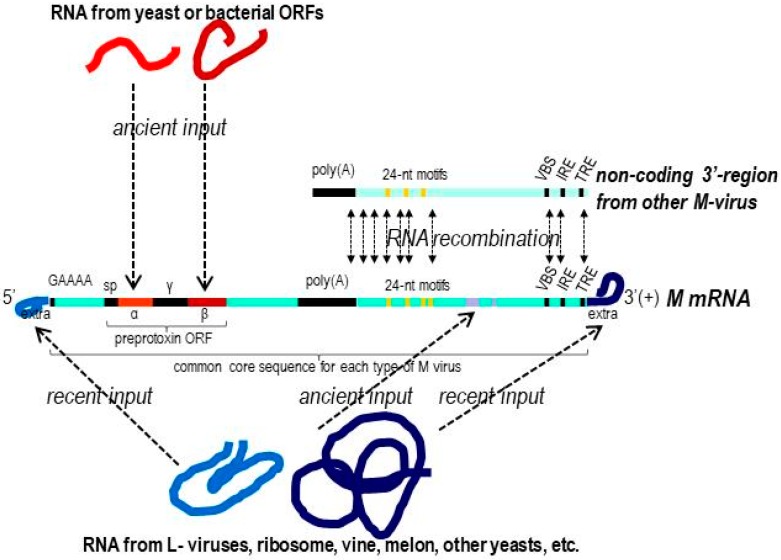
Model to explain the phylogenetic origin of the different types and isotypes of M-viruses, the existence of a common organization of their genomes, and the absence of relevant overall sequence identity. Elements of the common genome organization (conformational frame) required for genome replication and translation, processing, and secretion of active killer toxin are shown as black lines; α and β, subunits of the mature killer toxin; γ, central polypeptide removed during preprotoxin processing; sp, signal peptide. The conserved 5′-GAAAA sequence and central poly(A) of M-genome (cDNA) are shown. VBS, viral binding site; IRE, internal replication enhancer; TRE, 3′-terminal recognition element. The common core sequence is conserved in all viruses belonging to the same M-virus type. Distinct dsRNA isotypes differ from each other in the 5′- and 3′-extra sequences.

The identity found in some sequence stretches located in the non-coding 3′-region of M genomes from *Saccharomyces*, *Z. bailii*, and *T. delbrueckii* (Figure 3A–C) seems to indicate that these viruses share a common phylogenetic origin [1]. Another possibility is that the phylogeny of these different virus types shares similar recombination events that incorporated highly identical sequences, but from different genomic origins, in their non-coding 3′-region. This is conceivable given that we found a 24-nt identical motif in the genome of several M viruses and several of the hosting yeasts. Additionally, some stretches located downstream from these motifs in Mlus and Mbarr viruses also shared relevant identity with genomic sequences of other organisms (*Vitis vinifera*, *Candida glabrata*, and *Torulaspora delbrueckii*). This indicates that RNA recombination of M virus and RNA present in the hosting yeasts or growth medium was also involved in the primary phylogenetic origin of these killer viruses (as illustrated in Figure 6).

Moreover, as this 24-nt motif is repeated five times in the Mzb genome and twice in the M1 genome (Figure 3B,C), one can suspect a RNA recombination between sequences of the same dsRNA molecule, between two dsRNA molecules of the same virus type, or between two dsRNA of different M virus types that may eventually co-infect the same yeast cell (Figure 6), as was the case for M1-1 and Mbarr-2B viruses in *T. delbrueckii* EX1257. This argument is reinforced by the fact that some M genomes shared some relevant identity in the stretch located between central poly(A) and the 24-nt motifs (Figure 4).

### 3.3. Sequence Comparison of the Killer Preprotoxins

Mlus, Mbarr, and M2 preprotoxins showed relevant identity with several yeast hypothetical proteins. All these sequence identities were found, or became more relevant, when only the C-terminal half of the proteins (killer preprotoxins and chromosomal hypothetical proteins) was considered (Figure 5). It seems like the C-terminal half of some yeast proteins was the original source of the amino acid sequence to construct the β-subunit of the mature killer toxins. The transfer of the chromosomal sequence from yeast to virus genome may have occurred by recombination between the yeast and the viral mRNAs, as previously suggested [1]. We found a similar situation for M28 preprotoxin, which showed identity with a *Lachancea lanzarotensis* ORF (LALA0S04e08240g1_1) of unknown function, but this time, the identities were more relevant in the N-terminal half of K28 toxin (data not shown). This indicates that the α-subunit of the killer toxin could also originally have come from yeast chromosomal genes (Figure 6). In contrast with these findings, the M1 ORF should have had a different phylogenetic origin because it showed about 100% identity with bacteria, instead of yeast hypothetical proteins. In this case, the identical sequences were located in both N- and C-terminal stretches of K1 protoxin, which indicates that α- and β-subunits of mature K1 toxin may originally come from bacterial proteins (Figure 5D). This is a surprising result because bacteria are not known to be able to host M killer viruses.

No relevant identity was found between the very N-terminal or central stretch of preprotoxin with chromosomal ORFs from yeasts or bacteria. This indicates that the signal peptide and the central γ region of killer preprotoxins, that are processed to yield mature toxins containing only α and β subunits, would had to have come from somewhere else along the virus's evolutionary pathway.

## 4. Conclusions

The HTS techniques have allowed us to reliably detect and sequence dsRNA from yeast killer viruses. The partial sequence identity of the M viruses with nucleotide and amino acid sequences in available data banks suggests that they could have recombined with some RNA molecule at hand, such as other RNA viruses, host RNAs, or probably even free RNA from bacteria, as has been found for RNA viruses of plants [33]. As a result, these virus genomes are non-homologous chimeras that succeed in nature, as long as they share a common genome organization that is required for virus replication (with the help of LA virus) and keep some functional domains essential for translation, processing, and secretion of active killer toxins. This allows yeasts hosting M-virus to be able to kill other competitor sensitive yeasts. As long as this conformational frame was maintained as functional in the genome of M-viruses, the incorporation of sequences from yeasts or bacteria by RNA-recombination events could be at the origin of the different types of M-viruses, which have different core sequences. The viruses can thus in some way pick up sequences from yeast or bacteria proteins with a given biological activity that will be the basis for the mechanism of action of the new emergent killer toxins. The different isotypes for each M-virus type contain the same common core sequence with relatively few nucleotide changes, and they differ mostly in the extra RNA sequences that seem to be added in the 5′ and 3′ ends. These extra sequence additions may come from late RNA recombination events occurring after the appearance of each M-virus type (Figure 6). Given this possible origin of M-viruses, the overall sequence identity would not be a good tool with which to analyze the phylogenetic relationship of these viruses. Instead, novel approaches are needed for this purpose which would probably involve the analysis of the secondary structure of some common domains of the M-genomes, which in the future may be defined as the conformational setting of basic sequences in which the new sequences incorporated are fitted in by some type of promiscuous RNA recombination.

## 5. Materials and Methods

### 5.1. Yeast Strains and Media

The yeasts studied in this work were killer strains of *S. cerevisiae* and *T. delbrueckii* (Table 2). The killer strains EX231, EX436, EX1125, EX1160, and EX1257 were chosen for this study because they contain one to three new isotypes of M-dsRNA with an electrophoretic migration faster than the corresponding previously-studied M-virus. Additionally, some strains such as EX231, EX1160, and EX1257 show atypical killer phenotype (see below). All these yeasts are prototrophic strains isolated from spontaneous fermentations of grapes from vineyards located in the Extremadura region (southwestern Spain). The killer phenotype and the presence of viral dsRNA (L and M) in two of these yeast strains have been analyzed previously: EX436 (Klus containing LA and Mlus dsRNA) and EX1180 (Kbarr-1 containing LA, LBC, and Mbarr-1 dsRNA). The nucleotide sequences of LA, LBC, and Mlus dsRNA from EX229 were previously determined by traditional techniques of cloning and sequencing [2,37,38], and that of Mbarr-1 from EX1180 by HTS techniques [1]. The industrial use of *T. delbrueckii* Kbarr yeasts is under patent application.

Standard culture media were used for yeast growth [39]. YEPD contained 1% yeast extract, 2% peptone, and 2% glucose. YEPD+cyh is YEPD supplemented with cycloheximide (cyh) to a final concentration of 2 µg/mL.

### 5.2. Determination of Yeast Killer Activity

Killer activity was tested on low-pH (pH 4.0 or 4.7) methylene blue plates (4 MB or 4.7 MB) [40] seeded with 100 μL of a 48-h grown culture of the sensitive strain [41]. Depending on the experiments, the strains being tested for killer activity were either loaded as 4 μL drops of stationary phase cultures, patched from solid cultures, or replica-plated onto the seeded MB plates. The plates were incubated for 4–8 days at 12 or 20 °C.

### 5.3. Total Nucleic Acid Preparation and Nuclease Digestion

The procedure for routine dsRNA and mitochondrial DNA (mtDNA) minipreps was described previously [1,42]. Digestion of DNA was done with DNAse I (RNAse-free, Fermentas Life Sciences, Sankt Leon-Rot, Germany) according to the manufacturer’s specifications. Digestion of RNA was performed with RNAse A (Sigma-Aldrich, Darmstadt, Germany) following the manufacturer’s indications. For selective degradation of single-stranded RNA, samples were incubated with RNAse A (10 µg/mL) in the presence of 0.5 M NaCl for 30 min at 37 °C. Samples were then processed through phenol/chloroform/isoamyl alcohol extraction to inactivate the enzyme before analysis by agarose gel electrophoresis [2].

### 5.4. Nucleic Acid Analysis for Killer Yeast Typing

The procedure for virus dsRNA analysis has been described previously [42]. The samples (4 μL) were directly separated in 1× TAE-1% agarose gel electrophoresis for virus dsRNA analysis. Nucleic acids were visualized on a UV transilluminator after ethidium bromide staining of the gels, and photographed with a Gel Doc 2000 (Bio-Rad, Hercules, CA, USA).

### 5.5. Viral dsRNA Purification

Total nucleic acid preparation from *S. cerevisiae* and *T. delbrueckii* strains was done by the procedures mentioned above [42]. L and M dsRNAs were obtained from each strain by CF-11 cellulose chromatography as described elsewhere [43], and further separated from other dsRNAs in the same strain by 1% agarose gel electrophoresis. Thereafter, the slower-moving dsRNA band (4.6–4.7 kb) and the faster-moving dsRNA bands (1.3–2.3 kb) were cut off from the gel and purified with RNaid^®^ Kit (MP Biomedicals, LLC, Illkrich, France), following the manufacturer’s indications. This procedure was repeated until more than 20 μg of each purified dsRNA had been obtained.

### 5.6. Preparation and Sequencing of cDNA Libraries from Purified Viral dsRNA

The purified dsRNA samples were sent to the Unidad de Genómica Cantoblanco (Fundación Parque Científico de Madrid, Madrid, Spain) for cDNA library preparation and high-throughput sequencing (HTS). Libraries from each purified band were prepared with the “TruSeq RNA Sample Preparation Kit” (Illumina, San Diego, CA, USA) following the company’s instructions, and using 200 ng of purified dsRNA as input (quantified with Picogreen). Briefly, this protocol started at the fragmentation step, skipping the RNA purification step as the viral dsRNA had previously been purified, as mentioned above. Thereafter, 15% DMSO was added to the Illumina fragment-prime solution before incubation at 94 °C for 8 minutes to facilitate dsRNA denaturation. The first strand of cDNA was synthesized using random primers, dTVN and dABN oligonucleotides (Isogen Life Science, De Meern, The Netherlands), and SuperScriptIII retrotranscriptase. The dTVN and dABN oligonucleotides were added to improve retrotranscription of the expected central poly(A) region of M viruses. Thereafter, the second cDNA strand synthesis, end repair, 3′-ends adenylation, and ligation of the TruSeq adaptors were done (Illumina, San Diego, CA USA). These adaptor oligonucleotides included signals for further amplification and sequencing, and also included short sequences referred to as indices, which allowed multiplexing in the sequencing run. An enrichment procedure based on PCR was then performed to amplify the library, ensuring that all molecules in the library included the desired adaptors at both ends. The number of PCR cycles was adjusted to 12, and the final amplified libraries were checked on a BioAnalyzer 2100 (Agilent Technology, Santa Clara, CA, USA). The libraries were denatured prior to seeding on a flow cell, where clusters were formed, and sequenced using 2 × 80–2 × 150 sequencing runs on a MiSeq instrument.

### 5.7. Viral dsRNA Sequence Assembly

The full-length genome virus sequences were reconstructed as follows: FastQC version 0.11.5 (http://www.bioinformatics.babraham.ac.uk/projects/fastqc) was used to analyze the sequence quality of FASTQ libraries, and Prinseq version 0.18.2 [44] to filter sequence reads with phred <28 (for this study we selected only high-quality reads), shorter than 50 bp, or presenting at least 5% of indeterminations (Ns) and trimming poly-N tails. Clean reads were de novo assembled using Spades [45] with k-mer sizes from 21 to 77. Subsequently, contigs shorter than 300 nucleotides were removed from the assembly file, and the remaining contigs were used as input to the non-redundant nucleotide database of the NCBI, and to a specific database of *Saccharomyces* viruses using the Blastn search of the NCBI Blast package [46]. The whole protocol of analyses was executed using the pipeline SeqAnnotation tool of the GPRO suite [47]. Then, the 5′ and the 3′ ends of each reconstructed viral genome were enlarged using a two-step strategy. First, a script designed ad hoc was used to identify from FASTQ libraries all possible reads whose ends form contigs of 100% identity with respect to the overlapping sequence (the authors can provide this script on request). Second, overlapping reads were aligned to the viral genome they correspond to using Geneious 6.0 [48]. The whole process was repeated a number of times (note that in each repetition both ends are extended and then new ends are created) until the ends were extended as much as possible. In order to verify and curate the final sequences with the extended ends, Bowtie2 [49] was used to map FASTQ libraries to the final sequences. Then, the IGV tool [50] was used to browse the BAM file reported by mapping and verify that coverage across the sequences was appropriate, and then export a consensus sequence in order to get a curated representative sequence from each viral genome.

### 5.8. Real-Time Quantitative PCR (qPCR) Conditions and Analysis

Each purified dsRNA sample (250 ng) was heated in the presence of DMSO and allowed to cool before reverse transcription with the High Capacity Transcription Kit (Applied Biosystems Inc, Foster City, CA, USA). Following reverse transcription, cDNAs were amplified using Pre-Amp polymerase (Applied Biosystems Inc, Foster City, CA, USA, 12 cycles, 5 nM primers), diluted ten-fold, and subjected to standard qPCR (LC480, Roche, Basel, Switzerland) using SYBR Green^®^ detection. Amplified products were analyzed for melting profiles and compared to negative controls, both NTCs and RTminus reactions consisting in reverse transcription of RNAs made in the absence of RT polymerase. Reactions were performed in triplicate, and were considered positive if *C*_t_ values were lower than 34.00 and melting curves showed a profile clearly distinguishable from negative controls. These analyses were performed by the Unidad de Genómica Cantoblanco (Fundación Parque Científico de Madrid, Madrid, Spain).

### 5.9. Miscellaneous

DNA manipulations (enzyme digestion, PCR, and electrophoresis) were done following standard literature methods [51]. Most of the enzymes were from Promega or Sigma. Synthetic oligonucleotides were from Biomers.

BLAST programs were used to search for similarities between the viral genomes or their ORF-encoded proteins and data bank nucleic acids or proteins. The ClustalW program was used for multiple sequence alignment [52], and the MFOLD program for prediction of folding and hybridization of ssRNA [53].

### 5.10. Nucleotide Sequence Accession Number

The ScV-M2-4 cDNA complete nucleotide sequence and the encoded M2-4 protein appear in the NCBI/GenBank under GenBank accession number MF957266.

## Figures and Tables

**Table 1:** Characteristics of the yeast strains and dsRNA M-virus genomes sequenced by HTS

Virus	Previous Estimated Size/Sequenced Length (bp)/Yeast Strain/ΔG	Newly Analysed Yeast Strain	Killer Phenotype/dsRNA Isotype	Size Estimated/Length (bp) Sequenced/ΔG	5′-Extra Sequence (bp)/% Identity with: Size [Position]	3′-Extra Sequence (bp)/% Identity with: Size [Position]	Stem-Loop Involving 5′-Extra Sequence [Position] (ΔG)	Stem-Loop Involving 3′-Extra Sequence [position] (ΔG)
M1	1830/1801/*S.c*. TF325/−1807	*S.c*. EX231	K1*/M1-1	1300/1933/−1937	66/94% *S.c*. LBC-2 virus, 66 nt [A(−)66 to T(−)1]	66 /no identity found	Not found	Not found
		*T.d*. EX1257	Kbarr-2/M1-2	1700/1933/−1916	66/no identity found	65/94% *S.c*. 26S rRNA, 77 nt [C1783 to G(+)57]	Not found	Not found
M2	1700/1163 + 209/*S.c*. 1384/−1861	*S.c*. EX88	K2/M2-3	1650/1625/−2020	33/no identity found	None	[A(−)23 to T126] (−69.5)	Not found
		*S.c*. EX1125	K2/M2-4	1750/1723/−2297	87/no identity found	44 /no identity found	[T(−)42 to A145] (−205)	[C1603 to G(+)39] (−137)
Mlus	2300/2033/*S.c*. EX229/−1970	*S.c*. EX229	Klus/Mlus-4	2300/2314/−2242	50/no identity found	230/98% *S.c*. 16S mit rRNA, 242 nt [C2022 to G(+)203]	[C(−)50 to G107] (−30.1)	Not found
		*S.c*. EX436	Klus/Mlus-1	2000/2346/−2409	51/93% *Vitis vinifera*, 47 nt [A(−)30 to T17], and 85% *Saccharomycopsis fibuligera*, 45 nt [A(−)30 to A15]	198/100% *S.c*. 18S rRNA, 198 nt [A(+)1 to G(+)198]	[C(−)51 to G107] (−46.0)	Not found
		*S.c*. EX1160	Klus*/Mlus-A	2100/2268/−2723	218/no identity found	17/no identity found	[T(−)125 to A97] (−246), or [A(−)67 to T129] (−62.8)	Not found
		*S.c*. EX1160	Klus*/Mlus-B	1700/1937/−2066	77/no identity found	None	Not found	Not found
		*S.c*. EX1160	Klus*/Mlus-C	1350/2055/−1962	22/no identity found	None	Not found	Not found
Mbarr-1	1700/1705/*T.d*. EX1180/−1727	*T.d*. EX1257	Kbarr-2*/Mbarr-2B	1300/1835/−1878	64/88% *Cucumis melo*, 41 nt [A(−)18 to G23]	66/97% *T.d*. (95% *S.c.*) 26S rRNA, 76 nt [G1759 to C(+)65]	[T(−)2 to A53] (−40.6)	Not found

ΔG was obtained for the ssRNA(+) with the program MFOLD. * Atypical killer phenotype. ΔG is in kJ/mol.

**Table 2:** Yeast strains used in this study.

Strain	Genotype [Relevant Phenotype]	Origin
*Sc* EX88 *	*MAT a/*α *HO/HO cyh^S^/cyh^S^* M2-3 [K2^+^]	M. Ramírez ^a^ (from wine)
*Sc* EX85 *	*MAT a/α* *HO/HO* *cyh^S^/cyh^S^* LA M2-3 [K2^+^]	M. Ramírez ^a^ (from wine)
*Sc* EX85R *	*MAT a/α HO/HO CYH^R^/cyh^S^* M2^0^ [cyh^R^ K2^0^]	M. Ramírez ^a^ (from EX85)
*Sc* EX229 *	*MAT a/α HO/HO cyh^S^/cyh^S^* LA LBC Mlus-4 [Klus^+^]	M. Ramírez ^a^ (from wine)
*Sc* EX229-R1 *	*MAT a/α HO/HO CYH^R^/cyh^S^* [cyh^R^ Klus^0^]	M. Ramírez ^a^ (from EX229)
*Sc* EX33 *	*MAT a/*α *HO/HO* [LA^0^ K1^0^ K2^0^ K28^0^ Klus^0^]	M. Ramírez ^a^ (from wine)
*Sc* EX73 *	*MAT a/*α *HO/HO* LA M2-3 [K2^+^]	M. Ramírez ^a^ (from wine)
*Sc* EX198 *	*MAT a/*α *HO/HO* LA LBC Mlus-3 [Klus^+^]	M. Ramírez ^a^ (from wine)
*Sc* EX231	*MAT a/*α *HO/HO* LA LBC M1-1 [K1^+^]	This study (from wine)
*Sc* EX436	*MAT a/*α *HO/HO* LA Mlus-1 [Klus^+^]	This study (from wine)
*Sc* EX1125	*MAT a/*α *HO/HO* LA LBC M2-4 [K2^+^]	This study (from wine)
*Sc* EX1160	*MAT a/*α *HO/HO* LA LBC Mlus-A Mlus-B Mlus-C[Klus^+^]	This study (from wine)
*Sc* F166 *	*MAT* α *leu1 kar1* LA-HNB M1 [K1^+^]	J.C. Ribas ^b^ (from R. Wickner)
*Sc* F182 *	*MAT* α *his2 ade1 leu2-2 ura3-52 ski2-2* LA M28 [K28^+^]	J. C. Ribas ^b^ (from M. Schmitt)
*Td* EX1180 *	*wt* LAbarr-1 Mbarr-1 [Kbarr-1^+^]	M. Ramírez ^a^ (from wine)
*Td* EX1180-11C4 *	*cyh^R^* LAbarr-1 Mbarr-1 [cyh^R^ Kbarr-1^+^]	M. Ramírez ^a^ (from EX1180)
*Td* EX1180-2K^−^ *	*cyh^R^* LAbarr-1 Mbarr-1^0^ [cyh^R^ Kbarr^0^]	M. Ramírez ^a^ (from EX1180)
*Td* EX1257	*wt* LAbarr-2 M1-2 Mbarr-2B [Kbarr-2^+^]	This study (from wine)
*Td* EX1257-CYH5	*cyh^R^* LAbarr-2 M1-2 Mbarr-2B [cyh^R^ Kbarr-2^+^]	M. Ramírez ^a^ (from EX1257)

* Strain used as standard for killer-phenotype plate assay; ^a^ M. Ramírez, Departamento de Ciencias Biomédicas, Universidad de Extremadura, Badajoz, Spain; ^b^ J. C. Ribas, Instituto de Biología Funcional y Genómica, CSIC/Universidad de Salamanca, Salamanca, Spain; *Sc*, *Saccharomyces cerevisiae*; *Td*, *Torulaspora delbrueckii*.

## Supplementary Materials

The following are available online at www.mdpi.com/2072-6651/9/9/292/s1, Figure S1: Attempt to cure Mbarr-2 killer virus with cycloheximide.
