# Supplementary material for: New Insights into the Genome Organization of Yeast Killer Viruses Based on “Atypical” Killer Strains Characterized by High-Throughput Sequencing
Source: Toxins (Basel). 2017 Sep 19;9(9):292. doi: 10.3390/toxins9090292 (PMC5618225; doi:10.3390/toxins9090292)
Supplement: Supplementary file 1 [file toxins-09-00292-s001.pdf]

Supplementary Materials: New Insights into the Genome Organization of Yeast Killer Viruses Based on “Atypical” Killer Strains Characterized by High-Throughput Sequencing

Manuel Ramírez, Rocío Velázquez, Antonio López-Piñeiro, Belén Naranjo, Francisco Roig and Carlos Llorens

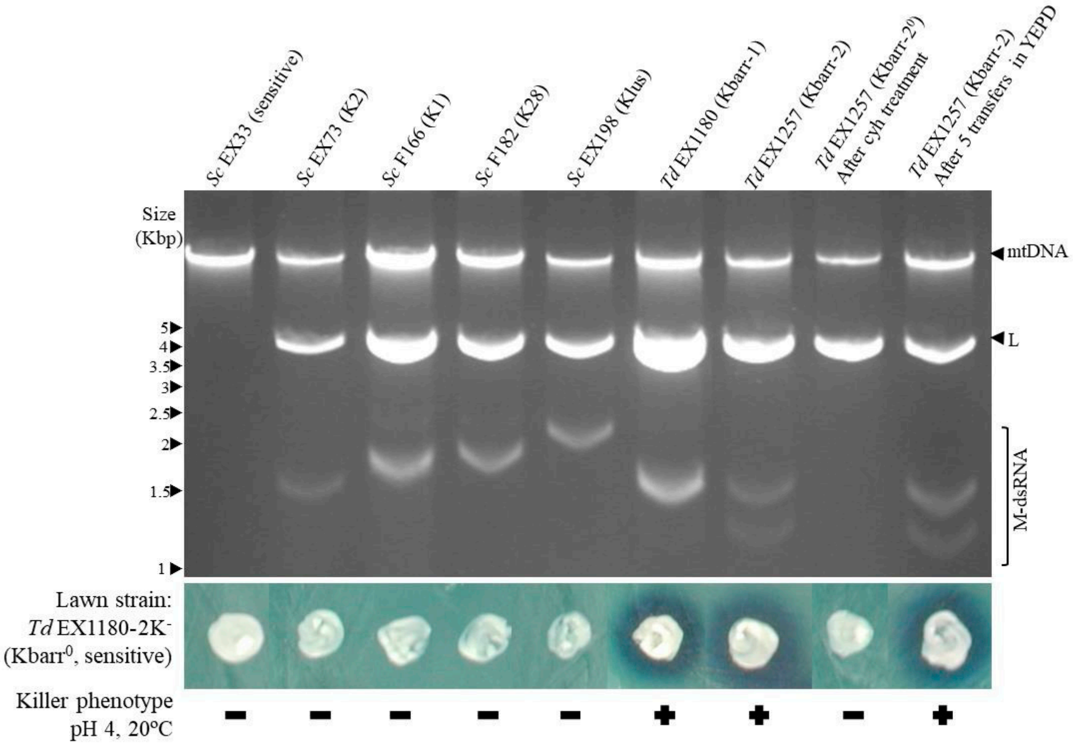

Figure S1. Attempt to cure Mbarr-2 killer virus with cycloheximide. Agarose gel electrophoresis of total nucleic acids from sensitive (EX33) and killer K2 (EX73), K1 (F166), K28 (F182), Klus (EX198), Kbarr-1 (EX1180), Kbarr-2 (EX1257), Kbarr-2^0 (EX1257 after cycloheximide treatment), and Kbarr-2 (EX1257 Kbarr-2^0 after cycloheximide treatment and 5 transfers in YEPD plates) strains (top panel). The killer phenotype assay is shown (bottom panel). EX1180-2K$^-$ is a cycloheximide-cured clone from EX1180. The assay was done on methylene blue agar plates (pH 4, 20 °C) seeded with *T. delbrueckii* EX1180-2K$^-$ strain.
